# Circulating Th1, Th2, Th9, Th17, Th22, and Treg Levels in Aortic Dissection Patients

**DOI:** 10.1155/2018/5697149

**Published:** 2018-09-06

**Authors:** Jing Ye, Yuan Wang, Zhen Wang, Qingwei Ji, Ying Huang, Tao Zeng, Haiying Hu, Di Ye, Jun Wan, Yingzhong Lin

**Affiliations:** ^1^Department of Cardiology, The People's Hospital of Guangxi Zhuang Autonomous Region, Nanning, China; ^2^Department of Cardiology, Hubei Key Laboratory of Cardiology, Renmin Hospital of Wuhan University, Cardiovascular Research Institute, Wuhan University, Wuhan 430060, China; ^3^Emergency & Critical Care Center, Beijing Institute of Heart, Lung, and Blood Vessel Diseases, Beijing Anzhen Hospital, Capital Medical University, Beijing 100029, China; ^4^Department of Ultrasound, The People's Hospital of Guangxi Zhuang Autonomous Region, Nanning 530021, China; ^5^Department of Cardiology, Handan First Hospital, Handan 056002, China

## Abstract

**Background:**

Previous studies demonstrated that the subsets of CD4+ T helper (Th) cells are closely related to vascular diseases, including atherosclerosis and hypertension. This study is aimed at investigating the circulating Th1, Th2, Th9, Th17, Th22, and Treg levels in aortic dissection (AD) patients.

**Methods:**

Blood samples from AD (*n* = 56) and non-AD (NAD, *n* = 24) patients were collected, and the circulating levels of Th1, Th2, Th9, Th17, Th22, and Treg cells and their transcription factors and functional cytokines were measured by flow cytometric analysis, quantitative polymerase chain reaction, and enzyme-linked immunosorbent assays, respectively. In addition, the human aortic vascular smooth muscle cells (HASMCs) were treated with saline, angiotensin II (Ang II), or plasma from AD patients.

**Results:**

Compared with the levels in the NAD group, the Th1, Th9, Th17, Th22, and their transcription factor levels were increased and the Th2, Treg, and their transcription factor levels exhibited a decreasing trend in AD patients. In addition, higher IFN-*γ*, IL-9, IL-17, and IL-22 levels and lower IL-4 and IL-35 levels were observed in AD patients. Simple linear regression analysis and binary logistic regression analysis suggested that Th1/IFN-*γ*, IL-9, Th17/IL-17, and Th22/IL-22 positively regulated the occurrence of AD, while Th2/IL-4 and Treg/IL-35 negatively regulated the occurrence of AD. Plasma from AD patients further increased Bax mRNA levels but decreased Bcl2 and *α*-SMA mRNA levels in Ang II-treated HASMCs.

**Conclusions:**

Changes in Th1, Th2, Th9, Th17, Th22, and Treg activity are associated with the onset of AD. Different subsets of CD4+ T cells play different roles in the presence of AD.

## 1. Introduction

Aortic dissection (AD) is a rare but dangerous clinical emergency that can result in extremely high mortality, especially when torn sections accumulate in the aortic arch [1]. Although the exact cause is unclear, abundant evidence has demonstrated that inflammatory cytokines play a critical role in the progression of AD [2–4].

Modern medicine divided CD4+ T helper (Th) cells into regulatory T cells (Treg) and effector T cells. To date, only one type of Treg has been found, which can protect against effector responses to autoantigens and harmful exogenous antigens. Effector T cells protect against pathogens and can be subdivided into several types according to their cytokine secretion profiles, including Th1, Th2, and Th17. In addition, Th22 and Th9, two novel subsets of effector T cells, were discovered in succession. Although each subset of CD4+ T cells can secrete a variety of cytokines and a cytokine can be secreted by several subsets of CD4+ T cells, each subgroup has characteristic inflammatory cytokines [5–9]. Th1, Th2, Th9, Th17, Th22, and Treg cells are involved in inflammatory responses and immune regulation mainly through the secretion of interferon- (IFN-) *γ*, IL-4, IL-9, IL-17, IL-22, and IL-35, respectively.

Previous studies demonstrated that CD4+ T cells and their functional cytokines are critical for vascular diseases. Knocking out IFN- and IL-17 and neutralizing IL-22 reduce elevated blood pressure in angiotensin (Ang) II-induced hypertension models [10–12]. Increased Th1/IFN-*γ*, Th17/IL-17, and Th22/IL-22 and decreased Th4/IL-4 were observed in human and mouse hypertension [12, 13]. In mouse atherosclerosis (AS), the Th1 immune response was demonstrated to increase the size of Ang II-induced AS [14]. Valsartan alleviated Ang II-induced AS via downregulating the Th2 response [15]. Both Th9 and Th22 responses aggravated high-fat diet-induced AS [16, 17]. The effect of Th17 responses on AS is controversial, as both aggravation and no effect have been reported [18–20]. In addition, higher Th1, Th2, Th9, Th17, and Th22 levels and lower Th2 and Th35 levels were observed in human coronary artery disease [21–24]. Previous studies found that Th17/IL-17 are involved in the progression of AD and plasma IL-22 levels were increased in human AD [25]. While the circulating Th1, Th2, Th9, Th17, Th22, and Treg levels in human AD remain unknown, this study is aimed at investigating these subsets of CD4+ T cells and their functional cytokines in human AD blood samples.

## 2. Materials and Methods

### 2.1. Collection and Processing of Human Blood Samples

The collection and processing of human blood samples from non-AD (NAD, *n* = 48) and AD (*n* = 56) patients were performed as described in our previous study [25, 26]. In brief, all the blood samples were collected after the computed tomography angiography (CTA) of the thoracic aorta was performed, but before the treatments. Patients who suffered from cardiovascular disease and other diseases which can affect the cardiovascular system were excluded from this study as our previous description [25, 26]. All blood samples were collected from the People's Hospital of Guangxi Zhuang Autonomous Region, and this study protocol was approved by the Medical Ethics Committee of the People's Hospital of Guangxi Zhuang Autonomous Region. The patients themselves or their families provided informed consent.

### 2.2. Flow Cytometry Analyses

Portions of the blood samples were collected into sodium heparin vacutainers (Becton Dickinson), and the peripheral blood mononuclear cells (PBMCs) were isolated in a Ficoll density gradient and resuspended in RPMI 1640 (Gibco) complete culture medium at a density of approximately 5 × 10^6^ cells/ml. Then, the PBMCs were treated with 2 *μ*l/ml cell stimulation cocktail in an environment with 5% CO_2_ at 37°C for 5 hours. The cells were collected and strained with fluorescein isothiocyanate (FITC) anti-human CD4 (FITC-CD4). The cells were treated with Fixation/Permeabilization Concentrate at room temperature for 60 min. Next, the cells were stained with phycoerythrin- (PE-) labeled anti-IFN-*γ* (PE-IFN-*γ*), PE-labeled anti-IL-4 (PE-IL-4), PE-labeled anti-IL-9 (PE-IL-9), PE-labeled anti-IL-17 (PE-IL-17), allophycocyanin- (APC-) labeled anti-IL-22 (APC-IL-22), or PE-labeled anti-CD25 (PE-CD25) + APC-labeled anti-Foxp3 (APC-Foxp3). Isotype controls were included for compensation and to confirm antibody specificity. Th1 cells were defined as CD4+IFN-*γ*+, Th2 cells were defined as CD4+IL-4+, Th9 cells were defined as CD4+IL-9+, Th17 cells were defined as CD4+IL-17+, Th22 cells were defined as CD4+IL-22+, and Treg cells were defined as CD4+CD25+Foxp3. Cell stimulation cocktails and all of the flow cytometry antibodies were purchased from eBioscience (California, USA) and used according to the manufacturer's instructions.

### 2.3. Enzyme-Linked Immunosorbent Assay (ELISA)

The plasma from each sample was thawed at room temperature, and plasma IFN-*γ*, IL-4, IL-9, IL-17, IL-22 (all from eBioscience), and IL-35 (Arigo) concentrations were measured using enzyme-linked immunosorbent assay (ELISA) kits according to the manufacturer's instructions.

### 2.4. Quantitative Polymerase Chain Reaction (RT-qPCR)

The blood cells were treated with TRIzol Reagent W, and the total mRNA was extracted. Then, cDNA was synthesized from 2 *μ*g of total mRNA using oligo (dT) primers and a reverse transcription kit according to the manufacturer's instructions. PCR amplifications were performed using LightCycler 480 SYBR Green Master Mix (all from Roche). The relative mRNA expression levels of T-bet, GATA3, PU.1, RORc, AHR, Foxp3, Bcl2, Bax, and *α*-SMA were measured, and the results were normalized against the expression levels of GAPDH. The RT-qPCR primer sequences are shown in Table 1.

### 2.5. Cell Culture

Human aortic vascular smooth muscle cells (HASMCs) were obtained from the National Infrastructure of Cell Line Resource (Beijing) and cultured in Dulbecco's modified Eagle's medium (DMEM; Gibco) which contained 10% fetal bovine serum (FBS) and 1% penicillin-streptomycin, at 37°C in a humidified atmosphere with 5% CO_2_. After a continuous passage, enough HASMCs were collected and planted into culture dishes. After being starved for 16 hours, the HASMCs were treated with saline, angiotensin II (Ang II, 100 nmol/l), and Ang II plus human plasma (10%) which was collected from AD patients. After treatment for 24 hours, the total RNA was collected from the HASMCs.

### 2.6. Statistical Analyses

First, whether the data conformed to the normal distribution was assessed. The mean ± standard deviation (SD) was used to present data with a normal distribution, and Student's *t*-tests were performed to analyze the differences between two groups. Medians (minimum-maximum) were used to present data with an abnormal distribution, and the Mann–Whitney *U* test was used to compare differences. The categorical variables were presented as counts (percentages) and compared with the chi-square test. Spearman's correlation was used to calculate correlations between plasma cytokine levels and CD4+ T cell percentages. To identify the effect of plasma cytokine levels and CD4+ T cells on the presence of AD, simple linear regression analyses and subsequent binary logistic regression analyses were performed. All the data were assessed by the SPSS 19.0 software (Chicago). A value of *p* < 0.05 was considered statistically significant.

## 3. Results

### 3.1. Basic Clinical Characteristics of the Patients

Compared with the values in the NAD group, higher fasting glucose (Glu), white blood cell (WBC), creatinine (CREA), D-dimer, and C-reactive protein (CRP) values were observed in the AD group. In contrast, no significant differences in gender, age, smoking, poor blood pressure control (PBPC), systolic blood pressure (SBP), diastolic blood pressure (DBP), total cholesterol (TC), total triglycerides (TG), high-density lipoprotein cholesterol (HDL-C), low-density lipoprotein cholesterol (LDL-C), heart rate (HR), time intervals between chest pain onset and the collection of blood samples, and medications were found between the NAD and AD groups. The clinical data for all patients are listed in Table 2.

### 3.2. Circulating Th1, Th2, Th9, Th17, Th22, and Treg Cells in AD Patients

**Figure 1:**
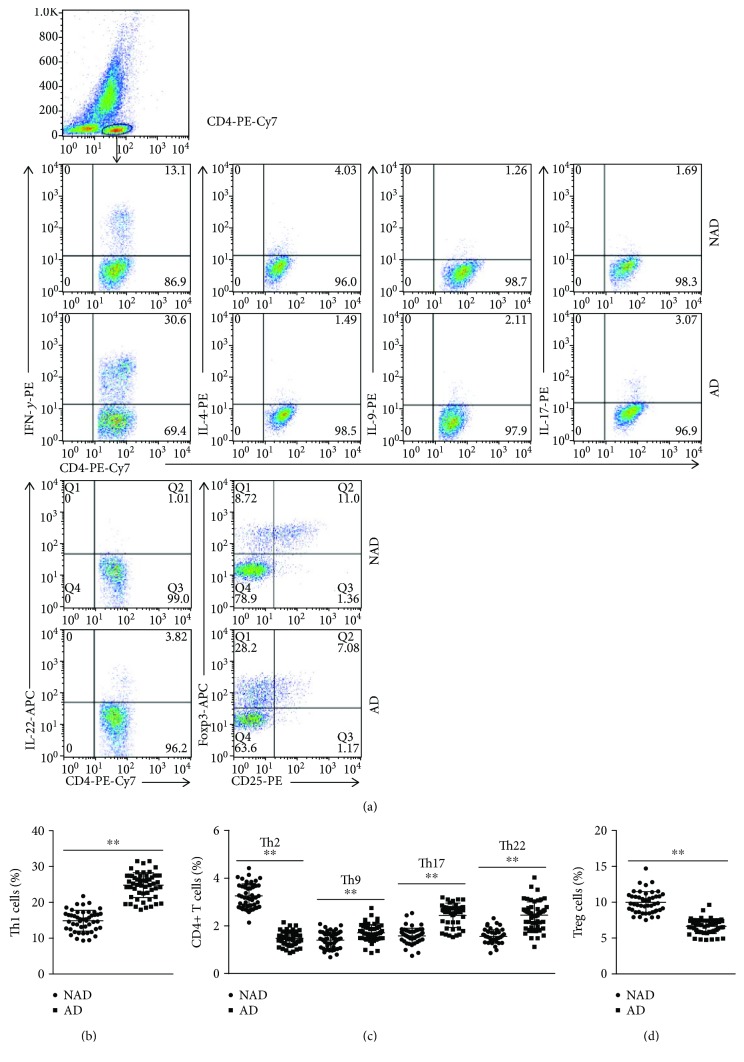
Circulating Th1, Th2, Th9, Th17, Th22, and Treg levels in NAD and AD patients. (a) CD4+ T cells were gated by flow cytometry, and intracellular cytokine staining of Th1, Th2, Th9, Th17, Th22, and Treg is represented for each group. (b–d) The frequencies of Th1, Th2, Th9, Th17, Th22, and Treg cells in the 2 groups. ^∗∗^*p* < 0.01 versus the NAD group.

**Figure 2:**
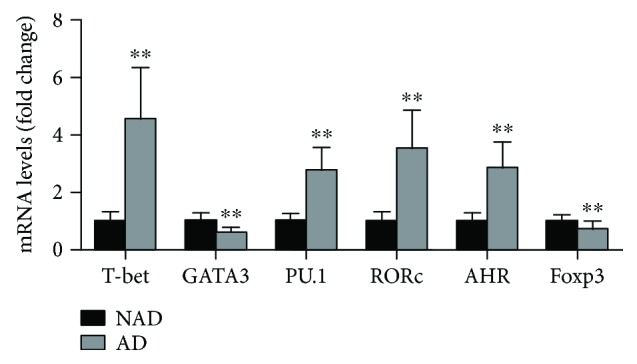
The mRNA levels of each subset of CD4+ T cell differentiation-required transcription factors in each group. T-bet, GATA3, PU.1, RORc, AHR, and Foxp3 mRNA levels in the NAD and AD groups. ^∗∗^*p* < 0.01 versus the NAD group.

**Figure 3:**
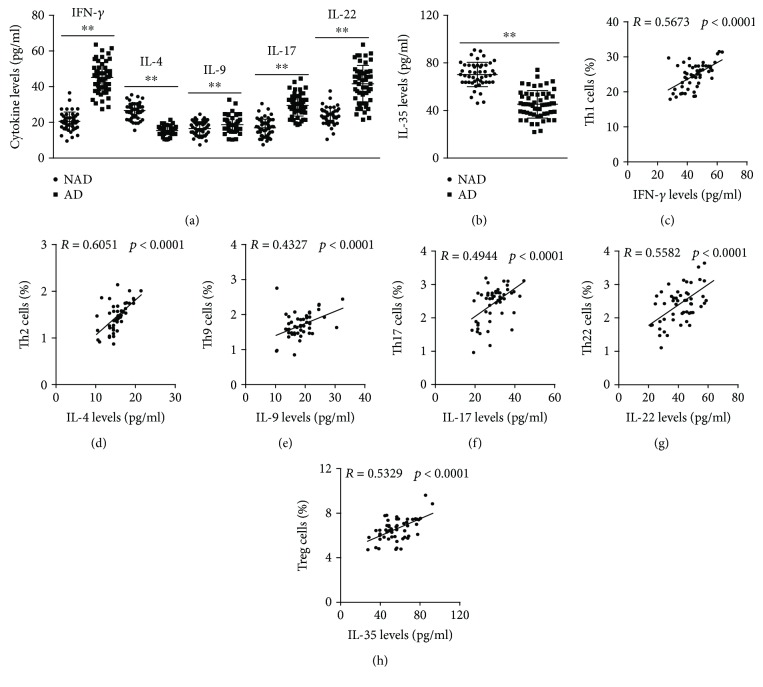
Plasma cytokine levels in each group. (a-b) IFN-*γ*, IL-4, IL-9, IL-17, IL-22, and IL-35 levels in the NAD and AD groups. (c–h) The correlation of Th1 cells and IFN-*γ*, Th2 cells and IL-4, Th9 cells and IL-9, Th17 cells and IL-17, Th22 cells and IL-22, and Treg cells and IL-35 in AD patients. ^∗∗^*p* < 0.01 versus the NAD group.

The circulating levels of each subset of CD4+ T cells were analyzed by flow cytometry, and the results showed that Th1, Th9, Th17, and Th22 levels were significantly increased in AD patients compared with the levels in NAD patients (Figures 1(a) and 1(c)), while lower Th2 and Treg levels were found in the AD group (Figures 1(a), 1(b), and 1(d)). In addition, we detected the mRNA levels of transcription factors required for T cell differentiation, and the same trend was found for these mRNA levels with these subsets of CD4+ T cells (Figure 2). To investigate the expression of the functional cytokines of CD4+ T cells, plasma IFN-*γ*, IL-4, IL-9, IL-17, IL-22, and Treg levels were measured by ELISA, and higher IFN-*γ*, IL-9, IL-17, and IL-22 levels and decreased IL-4 and IL-35 concentrations were observed in the AD group than in the NAD group (Figures 3(a) and 3(b)); this trend was consistent with the observed CD4+ T cell levels. We then assessed whether the CD4+ T cell levels were associated with their functional cytokines in AD patients, and the correlation analysis showed that circulating Th1, Th2, Th9, Th17, Th22, and Treg levels were positively correlated with plasma IFN-*γ*, IL-4, IL-9, IL-17, IL-22, and IL-35 concentrations, respectively (Figures 3(c)–3(h)). The percentages of each subset of CD4+ T cells and their functional cytokine concentrations are listed in Table 3.

### 3.3. The Role of Each Subset of CD4+ T Cells and Its Functional Cytokines in the Presence of AD

To investigate the roles of these subsets of CD4+ T cells and their functional cytokines in the presence of AD, simple linear regression analyses and subsequent binary logistic regression analyses were performed. Simple linear regression analyses showed that Glu, WBC, CRP, D-dimer, Th1, Th2, Th9, Th17, Th22, Treg, IFN-*γ*, IL-4, IL-9, IL-17, IL-22, and IL-35 levels exhibited a trend towards an association with the presence of AD, whereas smoking and CREA showed no obvious trend towards this association. These variables, including Glu, WBC, and CRP, were used to perform binary logistic regression analyses with the subsets of CD4+ T cell levels and their functional cytokines. The results suggested that Th1 (OR 0.175, 95% CI 0.068 to 0.282; *p* = 0.002), Th17 (OR 0.133, 95% CI 0.051 to 0.215; *p* = 0.002), and Th22 (OR 0.094, 95% CI 0.007 to 0.182; *p* = 0.035) levels were positively correlated with the occurrence of AD; Th2 (OR −0.437, 95% CI −0.555 to −0.319; *p* < 0.001) and Treg (OR −0.160, 95% CI −0.255 to −0.066; *p* < 0.001) levels were positively correlated with the occurrence of AD; and Th9 (OR −0.021, 95% CI −0.046 to 0.088; *p* = 0.541) levels had no correlation with the occurrence of AD (as shown in Table 4). On the other hand, IFN-*γ* (OR 0.303, 95% CI 0.205 to 0.402; *p* < 0.001), IL-9 (OR 0.072, 95% CI 0.013 to 0.130; *p* = 0.016), IL-17 (OR 0.095, 95% CI 0.011 to 0.179; *p* = 0.027), and IL-22 (OR 0.215, 95% CI 0.140 to 0.290; *p* < 0.001) levels were positively correlated with the occurrence of AD; while IL-4 (OR −0.222, 95% CI −0.318 to −0.125; *p* < 0.001) and IL-35 (OR −0.161, 95% CI −0.243 to −0.079; *p* < 0.001) levels were negatively correlated with the occurrence of AD (as shown in Table 5).

### 3.4. Effect of Plasma from AD Patients on Apoptosis of HASMCs

**Figure 4:**
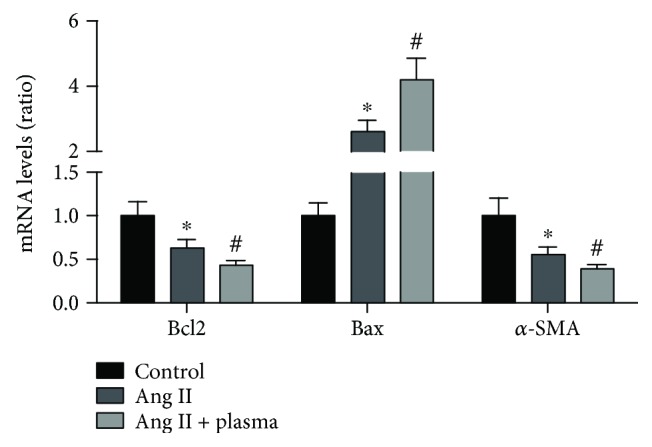
The mRNA levels of apoptosis gene and *α*-SMA in each group. The Bcl2, Bax, and *α*-SMA mRNA levels in the NAD and AD groups. ^∗^*p* < 0.05 versus the control group. ^#^*p* < 0.05 versus the Ang II group.

The mRNA levels of Bcl2, Bax, and *α*-SMA in these three groups were measured; the results showed that the Bax mRNA levels were significantly increased in the Ang II group and further increased when treated with plasma from AD patients; the opposite trend of mRNA levels of Bcl2 and *α*-SMA was observed (Figure 4).

## 4. Discussion

CD4+ T cells differentiate into several subsets, including Th1, Th2, Th17, Treg, and the recently discovered Th9 and Th22 cells. In previous studies, we, Ye et al., and Ju et al. found that changing IL-22 and Th17 levels were associated with the presence of AD [25, 27]. In this study, we detected all currently known subsets of CD4+ T cells and their functional cytokine expression, and we found for the first time that Th1/IFN-*γ*, Th2/IL-4, Th9/IL-9, Th17/IL-17, and Th22/IL-22 were increased in AD patients and positively associated with the presence of AD, while Th2/IL-4 and Treg/IL-35 were reduced in AD patients and negatively related to the presence of AD. In addition, treatment with plasma from AD patients increased HASMC apoptosis in vitro.

AD is a complicated degenerative disease of the aorta, and although many known causes can lead to the occurrence of AD, the specific mechanisms remain unknown. The infiltration of a large number of inflammatory cells, including neutrophils and macrophages, was found in human AD specimens and animal AD aortas [2–4, 25–27]. Gavazzi et al. and Liu et al. reported that reducing oxidative stress levels decreased the occurrence of AD [28, 29]. Significantly increased TUNEL cells and cleaved caspase-3, CHOP, and ATF4 levels were found in mouse and human AD aortas, while the knockout of CHOP reversed the levels of cleaved caspase-3 and TUNEL cells [30]. This evidence suggested that the overall inflammatory response, oxidative stress, autophagy, and apoptosis were critical for the presence of AD. Significant changes in the expression of all CD4+ T cell subsets were found in the present study, and our results further improved the inflammatory theory of AD.

Vascular smooth muscle cells (SMCs) are an important part of the aortic structure and play an important role in maintaining the normal structure and function of the aorta. A significant reduction of SMCs was observed in human and mouse AD aortic tissues, and this change was associated with AD progression [30]. In AD patients, ischemia and degeneration result in the necrosis of SMCs, and the myosin heavy chain of SMCs enters the blood circulation and begins to rise 3–6 hours after the onset of AD; the sensitivity and specificity of AD diagnosis within 12 hours were 90% and 97%, respectively [31, 32]. In addition, evidence demonstrated that AD is related to the degeneration of aortic media in pathology, characterized by the loss, fragmentation, and failure of SMCs [33]. Therefore, excessive loss of SMCs in the aorta is an important cause of AD morbidity. An excessive inflammatory response is one of the leading causes of excessive loss of SMCs because it can cause the apoptosis and excessive loss of SMCs in the aorta [30]. The effect of functional cytokines in the regulation of aortic SMC function and apoptosis was reported in previous studies [17, 34]. These lines of evidence suggested that the regulation of inflammatory responses and subsequent apoptosis of aortic SMCs was the possible mechanisms of CD4+ T cell involvement in the presence of AD, and more clinical experiments and animal studies are needed.

Chronic inflammatory responses are crucial for the progression of vascular diseases, including AS and abdominal aortic aneurysm (AAA). Although the specific mechanisms remain unclear, the successive imbalance of Th1/Th2 and Th17/Treg cells has been used to explain the pathogenesis of AS, although it cannot explain all the phenomena in the AS procession. In AAA, some evidence demonstrated that the Th1 immune response promotes the progression of AAA [35], while the Th2 immune response has an anti-AAA effect [36]. In addition, a variety of studies suggested that hyperactive Th17 immune responses promote the development of AAA [37], while Treg responses can inhibit the AAA process [38]. This evidence suggested that the imbalance of Th1/Th2 and Th17/Treg cells could also explain the mechanisms of AAA. Proinflammatory cytokines trigger immune disorders and amplify the inflammatory response in the circulation and blood vessels, leading to excessive loss of SMCs, vascular remodeling and dilation, and the development and rupture of AD. Anti-inflammatory cytokines can alleviate inflammatory responses, promote the repair of vascular tissue, and inhibit the presence of AD; therefore, inflammation is closely related to the presence of AD. Some subsets of CD4+ T cells and their functional cytokines play a proinflammatory role, while others play an opposing role. Our data demonstrated that increased Th1/IFN-*γ*, Th9/IL-9, Th17/IL-17, and Th22/IL-22 levels and decreased Th2/IL-4 and Treg/IL-35 levels are found in blood samples from AD patients. To investigate the roles of these subsets of CD4+ T cells in the presence of AD, simple linear regression analyses and subsequent binary logistic regression analyses were performed, and the results suggested that Th1/IFN-*γ*, Th9/IL-9, Th17/IL-17, and Th22/IL-22 levels were positively correlated with the occurrence of AD, while Th2/IL-4 and Treg/IL-35 levels were negatively correlated with the occurrence of AD. These data suggest that the imbalance of Th1/Th2 and Th17/Treg is one of the important mechanisms involved in the onset of AD, and rebalancing proinflammatory responses and anti-inflammatory responses is a new idea for the prevention and treatment of AD.

Previous evidence demonstrated that increased Th1/IFN-*γ*, Th17/IL-17, and Th22/IL-22 resulted in elevated blood pressure, and uncontrolled hypertension was one of the main reasons for the presence of AD because higher than normal blood pressure can lead to the apoptosis of vascular SMCs. In recent studies, Hayashi et al. reported that the downregulation induced by ultraviolet B irradiation significantly increased the rupture of angiotensin II-mediated aneurysms [39]. Honjo et al. also found that an ApoB-100-related peptide vaccine protects against angiotensin II-induced aortic aneurysm formation and rupture via decreasing Th17/IL-17 expression [40]. These lines of evidence indicated that CD4+ T cells are crucial to the rupture of aneurysms. Combined with previous studies, these findings indicated that CD4+ T cells could participate in a variety of vascular diseases, including AS, hypertension, AAA, and AD, and these vascular diseases have complex relationships with each other. In the clinic, many patients often suffer from more than one vascular disease, and the recovery of CD4+ T cells to normal levels is essential for the treatment of these patients.

Considering the importance of excessive loss of aortic SMCs due to apoptosis in the AD morbidity and the abnormal expression of the functional cytokines of CD4+ T cells in AD patients, a possible suspicion was that these abnormal expression cytokines may affect the excessive loss of aortic SMCs and participated in the occurrence of AD. To confirm this speculation, HASMCs were treated with plasma from AD patients and the HASMC apoptosis were detected; the results showed that plasma from AD patients increased Ang II-induced HASMC apoptosis and supported this speculation. While these results are the combined action of various cytokines, further researches are needed to investigate the special effect of each functional cytokine of CD4+ T cells on SMC apoptosis.

In conclusion, changes in circulating Th1, Th2, Th4, Th9, Th17, Th22, and Treg levels were found in AD patients. There are some limitations of this study. First, neutrophils, macrophages, and dendritic cells are important inflammatory cells, and we did not detect the levels of these cells. In addition, the sample sizes were small, and more patients are needed to validate the results.

## Figures and Tables

**Table 1:** RT-PCR primers used.

Gene	Forward primer	Reverse primer
T-bet	GTGACCCAGATGATTGTGCT	GGTTGGGTAGGAGAGGAGAG
GATA3	GAATGCCAATGGGGACCCTG	CTAACCCATGGCGGTGACCA
PU.1	CCAGCTCAGATGAGGAGGAG	CAGGTCCAACAGGAACTGGT
RORc	CCTGGGCTCCTCGCCTGACC	TCTCTCTGCCCTCAGCCTTGCC
AHR	CTTCCAAGCGGCATAGAGAC	AGTTATCCTGGCCTCCGTTT
Foxp3	CTGACCAAGGCTTCATCTGTG	ACTCTGGGAATGTGCTGTTTC
Bcl2	TCGCCCTGTGGATGACTGA	CAGAGACAGCCAGGAGAAATCA
Bax	TGGCAGCTGACATGTTTTCTGAC	TCACCCAACCACCCTGGTCTT
*α*-SMA	TGACAATGGCTCTGGGCTCTGTAA	TTCGTCACCCACGTAGCTGTCTTT
GAPDH	GAGTCAACGGATTTGGTCGT	GACAAGCTTCCCGTTCTCAG

**Table 2:** Clinical characteristics in patients who provide blood samples.

Characteristic	NAD	TAD	*p* value
Gender (M/F)	36/12	43/13	0.999
Age (years)	55 ± 8	58 ± 11	0.107
Smoking (*n*, %)	18 (37.5%)	24 (42.9%)	0.689
PBPC (*n*, %)	32 (66.7%)	43 (76.8%)	0.279
Glu (mmol/l)	6.2 (5.2, 7.9)	7.4 (6.1, 8.9)	0.003
SBP (mmHg)	156 (131, 165)	144 (130, 170)	0.784
DBP (mmHg)	81 ± 13	80 ± 15	0.888
WBC (×10^9^/l)	6.8 ± 1.2	10.1 ± 4.0	<0.001
TC (mmol/l)	4.2 (3.9, 4.9)	4.3 (3.8, 5.0)	0.417
TG (mmol/l)	1.5 (1.3, 2.0)	1.5 (1.1, 2.0)	0.891
HDL-C (mmol/l)	1.0 (0.9, 1.3)	1.0 (0.8, 1.4)	0.930
LDL-C (mmol/l)	2.2 ± 0.7	2.1 ± 0.7	0.648
HR (bpm)	81 (74, 92)	83 (72, 93)	0.586
CREA (*μ*mol/l)	76 (66, 89)	83 (71, 107)	0.017
D-dimer (*μ*g/ml)	0.6 (0.3, 1.0)	4.3 (2.3, 7.3)	<0.001
CRP (mg/l)	1.0 (0.4, 1.5)	19.0 (7.6, 77.6)	<0.001
Time (hour)	20 (12, 27)	24 (12, 34)	0.565
*Medications, n (%)*			
ACEI/ARB	24 (50.0%)	33 (58.9%)	0.431
Beta-blockers	9 (18.8%)	17 (30.4%)	0.256
CCB	23 (47.9%)	30 (53.6%)	0.552
Diuretic	11 (22.9%)	8 (14.3%)	0.313

PBPC: poor blood pressure control; Glu: fasting glucose; SBP: systolic blood pressure; DBP: diastolic blood pressure; WBC: white blood cell; TC: total cholesterol; TG: total triglycerides; HDL-C: high-density lipoprotein cholesterol; LDL-C: low-density lipoprotein cholesterol; HR: heart rate; CREA: creatinine; CRP: C-reactive protein; Time: time intervals between chest pain onset and collection of blood samples; ACEI: angiotensin-converting enzyme inhibitor; ARB: angiotensin receptor blocker; CCB: calcium channel blocker.

**Table 3:** CD4+ Th cells and their functional cytokine levels in NAD and AD groups.

Characteristic		NAD	AD	*p*
CD4+ Th cells (%)	Th1	15.0 (12.6–16.8)	25.4 (22.5–27.4)	<0.001
	Th2	3.18 (2.80–3.69)	1.48 (1.27–1.68)	<0.001
	Th9	1.43 (1.12–1.64)	1.67 (1.48–1.92)	0.001
	Th17	1.58 (1.44–1.77)	2.58 (2.14–2.75)	<0.001
	Th22	1.52 (1.42–1.70)	2.51 (2.12–2.70)	<0.001
	Treg	9.80 (8.83–10.8)	6.54 (5.86–7.46)	<0.001

Cytokines (pg/ml)	IFN-r	20.0 (17.5–23.4)	45.5 (38.8–50.1)	<0.001
	IL-4	27.2 (23.3–29.6)	15.5 (14.5–16.9)	<0.001
	IL-9	16.4 (13.9–19.6)	18.5 (15.8–21.4)	0.039
	IL-17	16.4 (12.8–20.4)	29.0 (25.8–32.5)	<0.001
	IL-22	24.2 (20.4–26.4)	43.0 (34.0–48.5)	<0.001
	IL-35	87.3 (79.7–97.5)	56.4 (46.6–67.1)	<0.001

**Table 4:** Association between CD4+ T cells and the presence of acute AD was assessed by simple linear regression analysis and subsequent binary logistic regression analysis.

Variables	Simple linear			Binary logistic		
	*β*	95% CI	*p* value	*β*	95% CI	*p* value
Th1	0.841	0.734 to 0.947	<0.001	0.175	0.068 to 0.282	0.002
Th2	−0.912	−0.992 to −0.831	<0.001	−0.437	−0.555 to −0.319	<0.001
Th9	0.404	0.224 to 0.584	<0.001	−0.021	−0.046 to 0.088	0.541
Th17	0.710	0.572 to 0.848	<0.001	0.133	0.051 to 0.215	0.002
Th22	0.702	0.562 to 0.842	<0.001	0.094	0.007 to 0.182	0.035
Treg	−0.805	−0.921 to −0.688	<0.001	−0.160	−0.255 to −0.066	<0.001
Glu	0.254	0.064 to 0.444	0.009	0.046	−0.018 to 0.110	0.156
WBC	0.473	0.300 to 0.646	<0.001	−0.011	−0.283 to 0.065	0.778
CRP	0.531	0.365 to 0.697	<0.001	0.062	−0.013 to 0.136	0.103
D-dimer	0.604	0.448 to 0.761	<0.001	0.060	−0.020 to 0.139	0.139
Smoking	0.054	−0.142 to 0.251	0.583			
CREA	0.155	−0.039 to 0.349	0.117			

**Table 5:** Association between CD4+ T cell-related cytokines and the presence of acute AD was assessed by simple linear regression analysis and subsequent binary logistic regression analysis.

Variables	Simple linear			Binary logistic		
	*β*	95% CI	*p* value	*β*	95% CI	*p* value
IFN-*γ*	0.867	0.770 to 0.965	<0.001	0.303	0.205 to 0.402	<0.001
IL-4	−0.853	−0.955 to −0.750	<0.001	−0.222	−0.318 to −0.125	<0.001
IL-9	0.232	0.041 to 0.423	0.018	0.072	0.013 to 0.130	0.016
IL-17	0.753	0.624 to 0.882	<0.001	0.095	0.011 to 0.179	0.027
IL-22	0.739	0.607 to 0.871	<0.001	0.215	0.140 to 0.290	<0.001
IL-35	−0.754	−0.883 to −0.625	<0.001	−0.161	−0.243 to −0.079	<0.001
Glu	0.254	0.064 to 0.444	0.009	0.044	−0.017 to 0.105	0.157
WBC	0.473	0.300 to 0.646	<0.001	−0.025	−0.093 to 0.044	0.479
CRP	0.531	0.365 to 0.697	<0.001	0.077	−0.009 to 0.144	0.027
D-dimer	0.604	0.448 to 0.761	<0.001	0.113	0.041 to 0.186	0.003
Smoking	0.054	−0.142 to 0.251	0.583			
CREA	0.136	−0.039 to 0.349	0.117			

## Data Availability

The authors declared that materials described in the article, including all relevant raw data, will be freely available to any scientist wishing to use them for noncommercial purposes, without breaching participant confidentiality.

## References

[1] Hagan P. G., Nienaber C. A., Isselbacher E. M. (2000). The International Registry of Acute Aortic Dissection (IRAD): new insights into an old disease. *JAMA*.

[2] Anzai A., Shimoda M., Endo J. (2015). Adventitial CXCL1/G-CSF expression in response to acute aortic dissection triggers local neutrophil recruitment and activation leading to aortic rupture. *Circulation Research*.

[3] Son B. K., Sawaki D., Tomida S. (2015). Granulocyte macrophage colony-stimulating factor is required for aortic dissection/intramural haematoma. *Nature Communications*.

[4] Zhang P., Hou S., Chen J. (2016). Smad4 deficiency in smooth muscle cells initiates the formation of aortic aneurysm. *Circulation Research*.

[5] Soon M. S. F., Haque A. (2018). Recent insights into CD4^+^ Th cell differentiation in malaria. *Journal of Immunology*.

[6] Tumes D. J., Papadopoulos M., Endo Y., Onodera A., Hirahara K., Nakayama T. (2017). Epigenetic regulation of T-helper cell differentiation, memory, and plasticity in allergic asthma. *Immunological Reviews*.

[7] Lee G. R. (2018). The balance of Th17 versus Treg cells in autoimmunity. *International Journal of Molecular Sciences*.

[8] Dudakov J. A., Hanash A. M., van den Brink M. R. M. (2015). Interleukin-22: immunobiology and pathology. *Annual Review of Immunology*.

[9] Duggleby R., Danby R. D., Madrigal J. A., Saudemont A. (2018). Clinical grade regulatory CD4^+^ T cells (Tregs): moving toward cellular-based immunomodulatory therapies. *Frontiers in Immunology*.

[10] Kamat N. V., Thabet S. R., Xiao L. (2015). Renal transporter activation during angiotensin-II hypertension is blunted in interferon-*γ*^−/−^ and interleukin-17A^−/−^ mice. *Hypertension*.

[11] Madhur M. S., Lob H. E., McCann L. A. (2010). Interleukin 17 promotes angiotensin II-induced hypertension and vascular dysfunction. *Hypertension*.

[12] Ye J., Ji Q., Liu J. (2017). Interleukin 22 promotes blood pressure elevation and endothelial dysfunction in angiotensin II–treated mice. *Journal of the American Heart Association*.

[13] Ji Q., Cheng G., Ma N. (2017). Circulating Th1, Th2, and Th17 levels in hypertensive patients. *Disease Markers*.

[14] Mazzolai L., Duchosal M. A., Korber M. (2004). Endogenous angiotensin II induces atherosclerotic plaque vulnerability and elicits a Th1 response in ApoE^−/−^ mice. *Hypertension*.

[15] Meng K., Zeng Q., Lu Q. (2015). Valsartan attenuates atherosclerosis via upregulating the Th2 immune response in prolonged angiotensin II-treated *ApoE*^−/−^ mice. *Molecular Medicine*.

[16] Zhang W., Tang T., Nie D. (2015). IL-9 aggravates the development of atherosclerosis in ApoE^−/−^ mice. *Cardiovascular Research*.

[17] Rattik S., Hultman K., Rauch U. (2015). IL-22 affects smooth muscle cell phenotype and plaque formation in apolipoprotein E knockout mice. *Atherosclerosis*.

[18] Smith E., Prasad K. M. R., Butcher M. (2010). Blockade of interleukin-17A results in reduced atherosclerosis in apolipoprotein E-deficient mice. *Circulation*.

[19] Erbel C., Chen L., Bea F. (2009). Inhibition of IL-17A attenuates atherosclerotic lesion development in apoE-deficient mice. *Journal of Immunology*.

[20] Madhur M. S., Funt S. A., Li L. (2011). Role of interleukin 17 in inflammation, atherosclerosis, and vascular function in apolipoprotein e-deficient mice. *Arteriosclerosis, Thrombosis, and Vascular Biology*.

[21] Ding R., Gao W., He Z. (2015). Overrepresentation of Th1- and Th17-like follicular helper T cells in coronary artery disease. *Journal of Cardiovascular Translational Research*.

[22] Lin Y. Z., Wu B. W., Lu Z. D. (2013). Circulating Th22 and Th9 levels in patients with acute coronary syndrome. *Mediators of Inflammation*.

[23] Madhumitha H., Mohan V., Deepa M., Babu S., Aravindhan V. (2014). Increased Th1 and suppressed Th2 serum cytokine levels in subjects with diabetic coronary artery disease. *Cardiovascular Diabetology*.

[24] Lundberg A. K., Jonasson L., Hansson G. K., Mailer R. K. W. (2017). Activation-induced FOXP3 isoform profile in peripheral CD4+ T cells is associated with coronary artery disease. *Atherosclerosis*.

[25] Ye J., Wang M., Jiang H. (2017). Increased levels of interleukin-22 in thoracic aorta and plasma from patients with acute thoracic aortic dissection. *Clinica Chimica Acta*.

[26] Xu Y., Ye J., Wang M. (2018). Increased interleukin-11 levels in thoracic aorta and plasma from patients with acute thoracic aortic dissection. *Clinica Chimica Acta*.

[27] Ju X., Ijaz T., Sun H. (2013). Interleukin-6-signal transducer and activator of transcription-3 signaling mediates aortic dissections induced by angiotensin II via the T-helper lymphocyte 17-interleukin 17 axis in C57BL/6 mice. *Arteriosclerosis, Thrombosis, and Vascular Biology*.

[28] Gavazzi G., Deffert C., Trocme C., Schäppi M., Herrmann F. R., Krause K. H. (2007). NOX1 deficiency protects from aortic dissection in response to angiotensin II. *Hypertension*.

[29] Liu W., Wang B., Wang T. (2016). Ursodeoxycholic acid attenuates acute aortic dissection formation in angiotensin II-infused apolipoprotein E-deficient mice associated with reduced ROS and Increased Nrf2 levels. *Cellular Physiology and Biochemistry*.

[30] Jia L.-X., Zhang W.-M., Zhang H.-J. (2015). Mechanical stretch-induced endoplasmic reticulum stress, apoptosis and inflammation contribute to thoracic aortic aneurysm and dissection. *The Journal of Pathology*.

[31] Suzuki T., Katoh H., Tsuchio Y. (2000). Diagnostic implications of elevated levels of smooth-muscle myosin heavy-chain protein in acute aortic dissection. The smooth muscle myosin heavy chain study. *Annals of Internal Medicine*.

[32] Suzuki T., Katoh H., Watanabe M. (1996). Novel biochemical diagnostic method for aortic dissection. Results of a prospective study using an immunoassay of smooth muscle myosin heavy chain. *Circulation*.

[33] Nienaber C. A., Eagle K. A. (2003). Aortic dissection: new frontiers in diagnosis and management: part I: from etiology to diagnostic strategies. *Circulation*.

[34] Skowron W., Zemanek K., Wojdan K. (2015). The effect of interleukin-35 on the integrity, ICAM-1 expression and apoptosis of human aortic smooth muscle cells. *Pharmacological Reports*.

[35] Xiong W., Zhao Y., Prall A., Greiner T. C., Baxter B. T. (2004). Key roles of CD4^+^ T cells and IFN-*γ* in the development of abdominal aortic aneurysms in a murine model. *Journal of Immunology*.

[36] Uchida H. A., Kristo F., Rateri D. L. (2010). Total lymphocyte deficiency attenuates AngII-induced atherosclerosis in males but not abdominal aortic aneurysms in apoE deficient mice. *Atherosclerosis*.

[37] Sharma A. K., Lu G., Jester A. (2012). Experimental abdominal aortic aneurysm formation is mediated by IL-17 and attenuated by mesenchymal stem cell treatment. *Circulation*.

[38] Yodoi K., Yamashita T., Sasaki N. (2015). Foxp3^+^ regulatory T cells play a protective role in angiotensin II-induced aortic aneurysm formation in mice. *Hypertension*.

[39] Hayashi T., Sasaki N., Yamashita T. (2017). Ultraviolet B exposure inhibits angiotensin II-induced abdominal aortic aneurysm formation in mice by expanding CD4^+^Foxp3^+^ regulatory T cells. *Journal of the American Heart Association*.

[40] Honjo T., Chyu K. Y., Dimayuga P. C. (2015). ApoB-100-related peptide vaccine protects against angiotensin II-induced aortic aneurysm formation and rupture. *Journal of the American College of Cardiology*.

